# 

**Published:** 1882-10

**Authors:** 


### CONTENTS.

COMMUNICATIONS.

Reflex Lesions of the Mouth, Associated with Pregnancy.............. 469
Some Miscellaneous Reflections on Dentistry and Dentists............... 474
On the Filling and Treatment of the Permanent Molar Teeth between the Ages of Six and Fourteen years.......................... 480
On the Process of Absorption in Bone and Tooth Structure............ 484
On Nicotine, and Its Action Upon the Teeth........................ 496

SELECTIONS.

A New Phase of the Amalgam Question.................................... 500
The Cause of Education................................................ 506
Application of Peroxide of Hydrogen for Medicinal Purposes......... 508
Sulphurous Acid as a Disinfectant...................................... 510
Impure Water Makes Impure Ice........................................ 511

CORRESPONDENCE.

From Geo. Watt, Xenia.................................................. 512
From Felix, Cross Roads................................................ 524
From L. C. Ingersoll................................................... 516
From Joseph Richardson................................................ 517

EDITORIAL.

Light—Vision............................................................ 518
The Colleges............................................................ 520
A Reminder.............................................................. 521
Absence................................................................. 522
Died.................................................................... 522

